# Priorities and preferences for school-based mental health services in India: a multi-stakeholder study with adolescents, parents, school staff, and mental health providers

**DOI:** 10.1017/gmh.2019.16

**Published:** 2019-08-19

**Authors:** R. Parikh, D. Michelson, M. Sapru, R. Sahu, A. Singh, P. Cuijpers, V. Patel

**Affiliations:** 1 Sangath, C-1/52, 1st Floor, Safdarjung Development Area, New Delhi, Delhi, India; 2 Department of Clinical, Neuro and Developmental Psychology, Faculty of Behavioural and Movement Sciences, Vrije Universiteit Amsterdam, van der Boechorstraat 1, Amsterdam, The Netherlands; 3 School of Psychology, University of Sussex, Falmer, Brighton, UK; 4 Evalueserve.com Private Limited, Tower 6, 8th Floor, Candor Gurgaon One Realty Projects Pvt. Ltd., IT/ITES SEZ, Candor TechSpace, Tikri, Sector-48, Gurgaon, Haryana, India; 5 International Rescue Committee, No 69/54, Oat Tha Phaya Street, Kyaikkasan Quarter, Bahan Township, Yangon, Myanmar; 6 Department of Clinical, Neuro and Developmental Psychology, Amsterdam Public Health Research Institute, Vrije Universiteit Amsterdam, van der Boechorstraat 1, Amsterdam, The Netherlands; 7 Department of Global Health and Social Medicine, Harvard Medical School, 641, Huntington Avenue, Boston, MA, USA

**Keywords:** Adolescents, India, qualitative, schools, stakeholder analysis

## Abstract

**Background.:**

Schools are important settings for increasing reach and uptake of adolescent mental health interventions. There is limited consensus on the focus and content of school-based mental health services (SBMHSs), particularly in low-resource settings. This study elicited the views of diverse stakeholders in two urban settings in India about their priorities and preferences for SBMHSs.

**Methods.:**

We completed semi-structured interviews and focus group discussions with adolescents (*n*  =  191), parents (*n*  =  9), teachers (*n*  =  78), school counsellors (*n*  =  15), clinical psychologists/psychiatrists (*n*  =  7) in two urban sites in India (Delhi and Goa). Qualitative data were obtained on prioritized outcomes, preferred content and delivery methods, and indicated barriers.

**Results.:**

All stakeholders indicated the need for and acceptability of SBMHSs. Adolescents prioritized resolution of life problems and exhibited a preference for practical guidance. Parents and teachers emphasized functional outcomes and preferred to be involved in interventions. In contrast, adolescents' favored limited involvement from parents and teachers, was related to widespread concerns about confidentiality. Face-to-face counselling was deemed to be the most acceptable delivery format; self-help was less frequently endorsed but was relatively more acceptable if blended with guidance or delivered using digital technology. Structured sensitization was recommended to promote adolescent's engagement. Providers endorsed a stepped care approach to address different levels of mental health need among adolescents.

**Conclusion.:**

SBMHSs are desired by adolescents and adult stakeholders in this setting where few such services exist. Sensitization activities are required to support implementation. School counsellors have an important role in identifying and treating adolescents with different levels of mental health needs, and a suite of interventions is needed to target these needs effectively and efficiently.

## Introduction

Mental health problems are a leading cause of the disease burden in adolescents. This burden is especially significant in low- and middle- income countries (LMICs) where adolescents constitute a high proportion of the total population. Mental health risks are abundant, and resources are scarce. Schools have been increasingly promoted in global efforts to reduce mental health disparities (Patton *et al*. 2016; World Health Organization, 2017). Educational environments exert a significant influence on adolescents' well-being, thereby offering unique opportunities to address the determinants of mental health (Kieling *et al*. 2011). In addition, school-based service provision can increase access to suitable interventions over and above existing mental healthcare systems, which are typically fragmented, under-resourced, and unsuited for adolescents' specific needs and preferences (World Health Organization, 2018).

India is home to the world's largest population of young people, comprising 20% of the total global population of adolescents. The pooled prevalence of mental health morbidity among adolescents in India has been estimated at 7.3%. This is likely to be much higher (13.3%) in urban metropolitan areas (Gururaj *et al*. 2016). India has one of the highest youth suicide rates globally and suicide is the leading cause of death in Indian youth (Patel *et al*. 2012). While no specific data are available for adolescents, the overall treatment gap for mental disorders in India is formidably high at around 90% (Gururaj *et al*. 2016).

To date, school mental health provision in India has generally been implemented on a relatively limited and fragmented scale, despite adolescent mental health being a priority area for service development in the National Adolescent Health Program (‘Rashtriya Kishor Swasthya Karyakram’; RKSK) (Ministry of Health & Family Welfare, 2015). Where available, SBMHSs have emphasized health-promoting school environments and fostering life skills of students (Srikala & Kishore Kumar, 2010; Kumar *et al*. 2015; Gujar & Pingale, 2017; Shinde *et al*. 2018). However, the wider literature on SBMHSs, primarily from high-income countries, suggests an expanded scope. This includes: (1) evidence-based promotive, preventive, and therapeutic interventions; (2) a stepped approach that organizes interventions according to different levels of need and resource constraints; (3) involvement of gate-keepers such as school administration, parents, and teachers in the development and delivery of the interventions; and (4) partnerships with local healthcare agencies for treatment of severe cases (Rones & Hoagwood, 2000; Langley *et al*. 2010; Kutcher *et al*. 2015; Murray & Jordans, 2016; Doll *et al*. 2017; Kern *et al*. 2017).

For a successful translation of these recommendations to India and other low-resource settings, it is essential for intervention developers and providers to understand and respond to local contexts, expectancies of relevant stakeholder groups, and anticipated barriers to implementation. The current study uses qualitative methods to explore the priorities and preferences of key stakeholders (adolescents, teachers, parents, and mental health providers) for the design of putative SBMHSs in two urban areas in India. This study was completed during the preliminary formative phase of ‘PRemIum for aDolEscents’ (PRIDE), a five-year research program which aims to develop and evaluate a contextually appropriate and scalable school-based intervention model for addressing an array of common adolescent mental health problems in India (Sangath, 2017). Initiated in 2016 at the field sites in Goa and Delhi, PRIDE builds on the Program for Effective Mental Health Interventions in Under-resourced settings (PREMIUM; 2010–2015), which established a systematic methodology for designing psychological interventions in LMICs (Vellakkal & Patel, 2015). PREMIUM focused on psychological treatments for adult depression (Healthy Activity Programme; HAP) and alcohol use disorders (Counselling for Alcohol Problems; CAP), involving extensive formative research and pilot studies, followed by definitive trials of both interventions (Nadkarni *et al*. 2017; Patel *et al*. 2017). PRIDE applied a similar developmental process in order to specify and optimize a transdiagnostic adolescent mental health intervention. The key formative sources were: (1) intervention design workshops with local and international experts; (2) scoping literature reviews; (3) ‘relevance mapping’ of evidence-based practice elements that can be applied most widely to presenting problems in the target population; and (4) local stakeholder interviews (Michelson *et al.* in press). The current study and a complementary report on context-specific stressors and coping strategies (Parikh *et al*. 2019) drew on a large volume of qualitative data collected from local stakeholder interviews (described above) during January 2016–May 2017. This allowed for triangulation of sources, leading to the specification of an intervention ‘blueprint’ followed by iterative field testing of intervention prototypes (Michelson *et al.* in press). The program will culminate in a series of randomized controlled trials (Parikh *et al*. in press).

## Methods

### Design and setting

A qualitative cross-sectional design was used to acquire a detailed and in-depth understanding of stakeholders' perspectives on SBMHSs in India. The study was conducted in Delhi (India's capital) and Goa (the country's most urbanized state) (Office of the Registrar General & Census Commissioner India, 2011), corresponding to research hubs managed by the implementing Indian organization (Sangath NGO). In Delhi, we recruited participants from eight Hindi-medium, single-gender high schools (grades 6–12) run by the Delhi Government in low-income areas; and one English medium, co-educational private school in a middle-class area. In Goa, we recruited participants from seven high schools (grades 5–10) run by the Archdiocese Board of Education. These schools provided co-education in Konkani and English languages in middle-class localities. The methods have been reported in line with the consolidated criteria for reporting qualitative studies (COREQ; see online Supplementary File 1) (Tong *et al*. 2007).

### Sample

Qualitative data collection focused on four stakeholder groups that were identified in preliminary scoping work as key to understanding priorities and preferences for SBMHSs in the study sites. These groups comprised (i) secondary school pupils; (ii) their parents; (iii) educational staff (teachers and principals); and (iv) mental health providers (school counsellors, clinical psychologists, and psychiatrists). Representatives from the various stakeholder groups were purposively sampled to achieve maximum variation across sites, age groups, and gender distribution (Table 1). In total, we collected qualitative data from 191 school-going adolescents (aged 11–17 years) through 22 focus group discussions (FGDs); 75 teachers through nine FGDs; three school principals, nine parents, five psychiatrists, and two clinical psychologists through in-depth interviews (IDIs); and 15 school counsellors through one FGD (10 school counsellors) and five IDIs. The final sample size was determined by saturation of themes emerging from the periodic analysis of detailed interview notes. Fewer parents were interviewed than we anticipated due to difficulties in making contact and scheduling interviews around other commitments (e.g. daily waged work in this mostly lower socio-economic sample). Adolescents were invited to participate in the study through classroom announcements and contacts with community-based organizations engaged with students from the participating schools. Other stakeholders were approached individually or in groups. Members of the same stakeholder group and adolescents of similar age (within 3 years) were grouped together in FGDs. Of the 22 FGDs conducted with adolescents, 12 (11 in Delhi; one in Goa) were single-gendered groups. The larger number of single-gendered groups in Delhi reflected the absence of co-educational schools in the local sampling frame.

**Table 1.** Sample characteristics of the participants of this study and site of data collection

Stakeholders	Delhi (*N*)	Goa (*N*)
Adolescents with mean age between 11 and 14 years (14 FGDs)	7 FGDs with 71 adolescents (boys = 18; girls = 53) 5 FGDs were conducted in schools and 2 FGDs were conducted at local community sites	7 FGDs with 51 adolescents (boys = 26; girls = 25) All FGDs were conducted in schools
Adolescents with mean age between 15 and 17 years (8 FGDs)	5 FGDs with 37 adolescents (boys = 17; girls = 20) 4 FGDs were conducted in schools and 1 FGD was conducted at a local community site	3 FGDs with 32 adolescents (boys = 18; girls 14) All FGDs were conducted in schools
Parents (9IDIs)	2 IDIs (male = 1; female = 1) Both parents were interviewed in schools	7 IDIs (male = 1; female = 6) 4 parents were interviewed in schools and 3 parents were interviewed at the office of the implementing Indian organization (Sangath NGO)
Teachers (9 FGDs, 3IDIs)	3 FGDs with 21 teachers (male = 1; female = 20) and 2 IDIs with school principals All teachers and principals were interviewed in schools	6 FGDs with 54 teachers (male = 18; female = 36) and 1 IDI with school principal All teachers and the principal were interviewed in schools
Mental health providers (12 IDIs and 1 FGD)	2 IDIs with psychiatrists, 2 IDIs with clinical psychologists and 3 IDIs with school counsellors Psychiatrists and clinical psychologists were interviewed in their clinics 2 school counsellors were interviewed in the schools and 1 in her office	3 IDIs with psychiatrists, 2 IDIs with school counsellors, and 1 FGD with 10 school counsellors Psychiatrists were interviewed in their clinics 2 school counsellors were interviewed in the school and 1 FGD was conducted in the department office of the school counsellors

IDI, in-depth interview; FGD, focus group discussion.

### Ethical considerations

Written informed consent was obtained from participants prior to data collection. For adolescents aged under 18 years, additional passive parent consent was also obtained (i.e. offering the choice for parents to opt-out from their own/their children's participation in the research). Approvals were obtained from the Institutional Review Boards at the Public Health Foundation of India (Ref:TRC-IEC-275/15), Sangath (Ref:VP_2015_017), Indian Council of Medical Research (Ref:HMSC/1/2016-SBR), and London School of Hygiene and Tropical Medicine (Ref:11967). Additional approvals were obtained from the Directorate of Education (Delhi) and Archdiocese Board of Education (Goa).

### Data collection

We collected data from each stakeholder group using semi-structured interview guides developed specifically for this study (online Supplementary File 2). Topics included prioritized outcomes, preferred content, and receptiveness to different modes of delivery for SBMHSs. The wording of questions was tailored to the specific stakeholder group, albeit with common themes reflected in all FGDs/IDIs. The topic guides also included questions related to common adolescent stressors and coping strategies, for which the corresponding data have been reported in another paper (Parikh *et al*. 2019).

Each FGD/IDI was conducted by one moderator and one note-taker over 45–60 minutes, at a convenient location (school, community, or office) (Table 1). Moderators included both males and females and held graduate, master's degrees in psychology or public health. All had prior training and experience in conducting qualitative interviews. All but three FGDs (adolescents  =  2; teachers  =  1) and two IDIs (school counsellor = 1; parents  =  1) were audio-recorded with prior permission of the participants and transcribed verbatim. The languages used for the FGDs and IDIs varied, such that raw qualitative data were obtained in English (*n*  =  34), Hindi (*n*  =  19), Marathi (*n*  =  1), and Konkani (*n*  =  2). Data analysis was undertaken in the original language, except for the Konkani transcripts which were first translated to English as none of the coders was fluent in Konkani. We analyzed detailed notes from the five FGDs that were not audio-recorded.

### Analysis

Thematic analysis was undertaken using an initial deductive coding framework based on four research questions: (1) What outcomes are prioritized by stakeholders with respect to SBMHSs? (2) What content is preferred by stakeholders in order to achieve these outcomes? (3) What delivery methods are endorsed by stakeholders? (4) What are the indicated barriers to implementation of SBMHSs? Next, three co-authors (RP, MS, and AS) independently coded 12 transcripts using Nvivo 11 software. Codes were compared, refined, and organized into thematic categories based on salient and prominent features of the data. The resultant coding framework was then applied to the remaining transcripts by RP, RS, and MS. Senior authors (DM and VP) reviewed themes and sub-themes at regular intervals to ensure coherence and relevance to the research questions.

## Results

Prominent themes are summarized below, supported by representative quotes from across the stakeholder groups. Quotes originally in Hindi, Marathi, and Konkani have been translated into English and marked with an asterisk (*). The gender and age group of quoted adolescents are indicated along with the relevant quotes.

### Prioritized outcomes

Most adolescents saw a need for SBMHSs to help with mitigating stress arising from a range of daily life challenges. The main stressors were pressure to perform in exams, anxiety about securing a job after education, one-sided romantic attractions, rejections and break-ups in romantic relationships, gaining peer acceptance, bullying, peer pressure, and family conflicts (often resulting from disagreements related to education and romantic relationships).

‘Some in class tease us with each other's names, or they will say that you are not good, some will go on to say that you are black and you are not fair… the girl or boy feel shy to talk with others or they will feel very sad that others are teasing us… if we go to the counsellor, then she will tell us not to feel shy, and we can behave as per her guidance… [and also get guidance] for dealing with bullies… or for household problems like problems with mother or aunt.’ (Girl, 14–15 years, Goa)

Parents prioritized outcomes related to improvements in ‘good’ and ‘obedient’ behaviors, which were closely related to parental expectations around exam performance and career prospects. Teachers focused on conduct problems around school attendance and classroom behavior, which were also strongly linked with academic attainment.

‘His behavior at home with everyone should be good and allow him to concentrate on study, because every parent wants their son to do well. And he is only one son to us, with 3 sisters. After growing-up he has to take their responsibility.’ (Parent, Delhi)*

‘If the students that are habitually late (at school), start coming on time and the ones who are lagging in studies, start scoring well in exams, or start attending school regularly… [this] would show that the students have improved.’ (Teacher, Delhi)*

School counsellors corroborated that students were likely to seek support with managing daily life stressors. Self-harm was prioritized as an additional target for girls across both the sites. Site-specific priorities also emerged from the school counsellors, including help for substance abuse among boys and Internet addiction for both boys and girls in Goa, and low self-esteem for both boys and girls in Delhi. Psychiatrists and psychologists highlighted the need to address diffuse stress and more focal anxiety, depression, anger, aggression/violent behavior, hyperactivity, substance use, and self-harm among adolescents across the sites. They also highlighted the significance attributed by parents and schools to declining academic performance as an indicator of poor mental health among adolescents.

‘I think in India, one of the main reasons for referrals is that the child is not performing in his school, or his academic deterioration, or he is not getting good grades, or not studying, refusing to study and then when we start exploring, we come to know that the person might be going through depression, [or] anxiety.’ (Psychologist, Delhi)

### Content of interventions

#### Coping strategies

Adolescents strongly endorsed practical advice, such as study tips to cope better with academic stress, and specific guidance to resolve interpersonal problems.

‘(Counselling) would help students to solve their problems, and you (counsellor) can provide such solutions… especially, if a student could not share his problems with anyone, he could share the problems with you… getting solutions would help students to become tension-free and they would not be scared anymore. They can freely mingle with everyone. They will be happy.’ (Girl, 14–16 years, Delhi)*

‘Helping them means to take them out from their depression. To help them with their issues and their problems. Suppose if we have problem and go for counselling then we feel good. We get the solution for it. The counsellor gives you the suggestion how to cope with your problems. We get more ideas from them to overcome our problems.’ (Girl, 12–15 years, Goa)

Mental health providers referred to specific techniques including problem-solving and life skills training to improve students' self-efficacy and problem-focused coping. The benefits of relaxation training were also highlighted by school counsellors, while psychiatrists and psychologists additionally recommended cognitive and behavioral techniques such as cognitive restructuring and behavioral activation, to enhance the adolescents' skills to cope with sadness, anxiety, and anger.

‘I think the program should include something which is common to all anxiety disorders, may be some components to deal with mood… how to deal with emotions like anxiety or sadness or anger.’ (Psychologist, Bangalore)

#### Psychoeducation

Information needs were emphasized across all stakeholder groups. Some adolescents sought to understand the causes and implications of mental health problems. A majority of participating parents and teachers preferred to be informed about the details of the index adolescent's presenting mental health problems, the likely causes and appropriate support that could be provided in the school and home settings. Psychologists and psychiatrists identified the need to tailor messages for different audiences using simple and non-stigmatizing language.

‘I would say for all 3 populations [adolescents, teachers, parents], some level of psychoeducation (is needed), which is tweaked to the needs as well as to the understanding and the psychological mindedness of that particular target group.’ (Psychologist, Delhi)

All stakeholders, but especially teachers and school counsellors, also noted the importance of providing school-wide and targeted information to generate awareness about the objectives and scope of SBMHSs, and thereby promote their uptake. School counsellors further highlighted the engagement of school administrators as being critical to the successful delivery of SBMHSs.

‘[Students need more information] that counselling is going on, like what do you mean by a counsellor, who is she?’ (Girl, 11–13 years, Delhi)*

‘And we have to create more awareness about this programs, because it is like some people feel, ‘why should I? I am mad or something like that?’ No! We have to open it up… Why not have a subject like that in the school as a part of syllabus… with once a week lecture, which will be nice for the children, so they will know where to come, where to go like that?’ (Parent, Goa)

‘So parents would have to be made aware about the mental health problems among adolescents. Only then can they identify these in their children and refer them. At present, I don't think they know much about this. Even as teachers, we too are not completely aware of the scope of mental health problems in adolescents.’ (Teacher, Delhi)*

### Delivery methods

#### Face-to-face counselling

Group-based interventions were rarely mentioned spontaneously. Nearly all adolescent participants interpreted questions about counselling in terms of individual formats, while occasional references were made to group formats in interviews with adult respondents. There were indications that group-based delivery might be helpful in terms of efficiency, but this was countered by concerns over the acceptability of group selection and delivery.

‘The teacher should select the child (for counselling), as they know their behavior in class. But the child may feel hurt that I am being singled out for counselling. So there could be groups who get counselling regularly… That is also a problem because speaking in general (in a group) could be the problem… some children are like I don't want to speak in front of everyone.’ (Parent, Goa)*

‘Sometimes we do group sessions also so if there are two or three children who have the same problem… then we discuss the issues together… They come with their best friends mostly… They would come for a common problem like study-related, or career advice, but not to discuss personal issues… Then from those, whoever need more help, they will come individually.’ (School counsellor, Goa)

Overwhelmingly, however, individual counselling was endorsed by all stakeholder groups as a means to provide personalized and responsive psychological help. Adolescents particularly highlighted the relational aspects of individual counselling as part of its appeal. The ideal counsellor was considered to be a warm and approachable person with an empathic, non-judgmental attitude. S/he should demonstrate an ability to relate with adolescents, and would preferably be of the same gender and slightly older than the index adolescent.

‘A counsellor should be the person who is very friendly [which] means when we enter that room we should get that feeling that we can open up with the person or he should be like a friend, who talks like a friend, make us feel comfortable… [which] means we can tell our problems.’ (Girl, 12–15 years, Goa)

‘And the counsellor should speak with us with warmth… He should be so nice that every kid can talk with them… Like, [a counsellor] should have good feelings towards the child, [a counsellor] should be able to explain things well, and [a counsellor] should be able to understand his problem.’ (Boy, 12–13 years, Delhi)*

Adolescents also requested clear assurances about confidentiality in order to create a safe, non-judgmental space for sharing problems.

‘Some will not go because we see, she is like a stranger… They can be afraid about telling their problems. They won't feel comfortable to tell their problems to the counsellor, they feel that counsellor will tell someone else, and if it is a big problem, then they are afraid that she can tell our parents… So, first you have to see that he or she is believable or not [which] means that we can trust him or her, that she does not say our personal feelings to any other person.’ (Girl, 14–15 years, Goa)

Parents, teachers, and mental health providers also recognized the value of an independent counsellor with specific expertise in engaging adolescents.

‘The counsellor should be someone who can mix well with students, meaning students should not feel shy to meet the counsellor or embarrassed to share all their problems.’ (Parent, Goa)*

‘But there are all those parents who have tried everything and given up and now they want something else… There are many times that children share much better with another person rather than their parents.’ (Teacher, Goa)

#### Parent and teacher involvement

Closely related to confidentiality concerns, adolescents very commonly expressed strong reservations about the extent of parent and teacher involvement in therapeutic activities. Adolescents were concerned that other adults would not be supportive or understanding of their mental health problems, particularly in cases involving socially prohibited stressors, such as those associated with intimate relationships. Involvement of significant adults was only deemed acceptable in serious cases such as those involving victimization, abuse, violence, and/or self-harm.

‘Now for example, a girl, despite counselling… she keeps thinking that ‘I want to die,’ then parents need to be involved… If [a counsellor] thinks that this is beyond [them], then parents should be involved. But if [a counsellor] thinks that [they] can help the child, then parents should not be involved.’ (Girl, 16–17 years, Delhi)*

Parents and teachers were less circumspect about the need for confidentiality, and expected to be informed about the adolescents' participation and progress in school counselling. Mental health providers acknowledged this tension and explained the importance of taking consent from adolescents where possible.

‘We take their consent. Yeah, we tell [adolescents] also that if we have to call your parents we will always take your consent and we will ask you who's your preferred parent or significant adult. And then we call the parents.’ (School counsellor, Goa)

#### Self-help

Respondents rarely made spontaneous references to self-help approaches. Consequently, brief descriptions of self-help concepts and formats (e.g. printed workbooks and digital apps) were used to explore receptiveness in FGDs/IDIs with all participants except mental health providers. Adolescents raised concerns about the limits of using self-help materials in order to better understand mental health concepts and learn coping skills. It was felt that two-way feedback (provided by a putative counsellor) would be needed in order to improve understanding. Doubts were also raised by adolescents about the applicability of pre-fabricated self-help materials to diverse problems. These concerns were tempered by perceived advantages pertaining to increased confidentiality relative to face-to-face counselling. Digital formats (provided on mobile or tablet-based devices) held particular appeal.

‘Suppose he has a problem, and in that book it is different situation, and then he cannot ask question from that book. But, if I am talking with the counsellor, then I can directly ask the question.’ (Boy, 12–15 years, Goa)

‘With an app you don't worry [that] it actually would spread through a person like a counsellor who knows your parents, who you think might go and tell your parents. So you can just go to the app and type whatever you feel.’ (Girl, 13–17 years, Goa)

‘We can read the book, but it is boring, and if it is on a tablet, it would be interesting. And everyone can type mostly.’ (Boy, 15–17 years, Delhi)*

In practice, however, the relatively low rate of ownership for personal digital devices, particularly among students in Delhi, led many adolescents and parents to conclude that digital delivery would not be feasible. While adolescents could potentially borrow devices from parents or older siblings, they were wary about explaining the purpose of the self-help app and negotiating restrictions around private use. For similar reasons, some teachers also commented that printed self-help materials were more likely to be feasible and acceptable across the sites.

‘If we are given a mobile, and even if we are doing some useful thing… then others will say ‘we don't know to whom she is talking.’ And parents also will think same, and they might take it away. We need to explain to our parents that this is an app that we are using and not doing anything wrong. So parents also should be told about it.’ (Girl, 13–16 years, Delhi)

‘As a parent, definitely I am already fed up seeing [adolescents] with their mobiles so much. I would not like it [self-help app], that's what I am saying.’ (Teacher, Delhi)

Mental health providers, and particularly school counsellors, were generally more enthusiastic about the potential for self-help to support scaling up of SBMHSs. Digital formats were seen as having scope for personalization through self-assessments, tailoring of ‘pushed’ information and selection of content from a ‘personalized toolbox’ of coping strategies.

‘Let's not make it general, let it be specific and match [adolescents’ problem]. So in that case, if an individual, he wants an information regarding bullying, I think videos can actually help.’ (School counsellor, Delhi)

#### Blended approaches

Adolescents identified a need for face-to-face guidance to clarify information and provide corrective feedback while using self-help materials. Mental health providers suggested additional roles for the guiding counsellor, including assessment, goal setting, establishing a therapeutic relationship, monitoring adherence and impact, managing risk, and making decisions about further treatment options. Although it was suggested that some guidance could be provided through emails, telephone calls, and text messages, there was a general consensus that self-help may be suboptimal unless accompanied by individual face-to-face interactions (i.e. as a blended approach). It was also proposed that guidance should be tailored for the age and comprehension level of the adolescent.

‘A younger child would need much more explanation to use it [self-help materials], the elder one will probably get it… I think the comprehension level would differ [across ages].’ (School counsellor, Goa)

‘As a therapist, I believe that my relationship with the client, which I have built, is the core of the work that we do and then other things kind of fall into place. So that is one thing that I see as a [disadvantage of self-help].’ (Psychologist, Delhi)

#### Multi-tiered approach to address different levels of need

All stakeholder groups recognized that adolescents would present with varying levels of mental health need in schools. Mental health providers additionally raised the prospect of employing a multi-tiered intervention framework incorporating universal and more targeted psychological support across different levels of need. Proposed examples of the former included life skills training, education about substance use, sexuality, and vocational guidance. It was suggested that indicated mental health prevention efforts (involving low-intensity formats) could focus on students with less severe, subthreshold presentations and time-limited adjustment difficulties, with individually tailored face-to-face therapy for students with more persistent and disabling problems. External referral to mental health specialists was recommended for adolescents with the most severe and high-risk presentations (e.g. self-harm and sexual abuse).

‘So, if you are going with the public health model then you will have to have a universal intervention for every child in the school.’ (Psychiatrist, Delhi)

‘If it is a school-based intervention for everybody, then you can have a uniform intervention wherein we have some common components. I think something basic that addresses most of the common problems and it is slightly guided by the counsellor, and once the intensity of the problem is high, it requires one-to-one therapy.’ (Psychiatrist, Bangalore)

### Potential barriers

Mental health stigma emerged most strongly as a barrier to service uptake in Goa, with mixed views noted in Delhi. Some adolescents in Goa schools had personally experienced or else observed teasing of peers who had engaged in counselling, whereas the provision of school counselling was a relatively novel prospect for most of the adolescents in Delhi. Mental health providers also noted that mental health stigma could vary widely between schools/settings.

‘When I first went to the counsellor, everyone thought that it must be related to boyfriend, everyone thinks that this could be the only reason why someone must see a counsellor… then they started making fun of me.’ (Girl, 14 years, Goa)*

‘Sir, there are many children here, who say that he is mad, he goes there (to counsellor), something is wrong with his mind, they tease.’ (Boy, 12–13 years, Delhi)*

‘When a child comes back from counselling, other children ask her to show what is written in the counselling book. She is considered to have some problem as she is going to counsellor. Others would read what is written in the book and make fun… and then she would feel that there is no point going.’ (Teacher, Goa)

Some teachers and mental health providers indicated that low awareness of mental health problems and their treatment among parents and teachers could lead to delays in seeking help. School counsellors from Goa additionally described challenges around engaging parents and teachers, explaining that significant adults often expected a ‘quick fix.’ Reflecting on prior experiences, counsellors felt that their credibility was undermined as a result of such unrealistic expectations.

‘In Goa, when Government started this scheme, we really had a tough problem. Some teachers were not keen, some teachers used to spoil counsellor's name [saying that] counsellor can't do anything, there is [not] much change. After one session they expected we should bring the change in the child's mind or the child's personality like a magician.’ (School counsellor, Goa)

School counsellors observed that many adolescents, especially if referred by teachers for behavioral problems, may be unmotivated to seek help and therefore fail to engage in an SBMHS. Other barriers, mentioned by teachers and parents across sites, included busy school schedules with little to no free time, such that counselling sessions might have to be scheduled in time slots that clashed with usual classes. Truancy was also seen as affecting the availability of students (especially the more troubled individuals) to attend sessions.

## Discussion

We conducted a multi-stakeholder analysis to understand priorities and preferences for school mental health provision in urban India. A key finding is that all stakeholder groups considered schools to be an acceptable platform for mental health service delivery. A variety of valued outcomes were identified: adolescents prioritized stress reduction as related to coping with life difficulties; parents and teachers prioritized functional outcomes (especially academic functioning); and mental health providers emphasized more overt psychiatric presentations. Site differences were also apparent, such that substance use and Internet addiction were highlighted as potential intervention targets in Goa, which can be inferred as reflecting differences in accessibility of substances and greater Internet connectivity in the Goan sample relative to peers in Delhi.

To date, SBMHSs in LMICs have primarily emphasized interventions for promoting adolescent mental health and improving school environment (Barry *et al*. 2013; Fazel *et al*. 2014; Kumar *et al*. 2015; Das *et al*. 2016); this study suggests that the scope of SBMHSs can be widened to include therapeutic interventions, tailored to the resources available and in line with the key preferences of stakeholders, most critically including adolescents themselves. In particular, all stakeholder groups endorsed psychoeducation (suggesting a key role in sensitization; see below) and problem solving as potential intervention elements. The latter reflects the primacy of psychosocial stressors in the adolescents' narratives about potential help-seeking; it is also among the most common elements of adolescent mental health interventions globally (Boustani *et al*. 2015), which further underlines its potential utility as a core component in SBMHSs. Mental health providers additionally suggested relaxation, cognitive restructuring, and behavioral activation, and endorsed a stepped care approach to providing interventions of differential intensity according to the type and severity of presentations. Although parents and teachers prioritized addressing self-harm for girls, the association of gender with suicidal attempts among young persons in India is less clear (Radhakrishnan & Andrade, 2012). Similarly, while substance use was more commonly referred to as a priority in boys in Goa, relatively high prevalence rates for substance use have been reported in many other parts of the country (Dhawan *et al*. 2017).

Adolescent respondents preferred a warm, relatable, empathic, and non-judgmental counsellor who ensured confidentiality, and provided a safe and private space for adolescents to share their problems. This is consistent with a large body of international research demonstrating the primary importance paid by young people (and service users more generally) to ‘non-specific’ relational aspects of mental health care (Butler, 2007; Persson *et al*. 2016; Van Os *et al*. 2019). Relatedly, studies from high-income countries have shown that face-to-face therapy may be preferred by adolescents over pure self-help and remotely delivered formats (Farrand *et al*. 2006; Bradford & Rickwood, 2014). In the current study, individual counselling was the most familiar and acceptable format outside the respondent group of mental health providers, whereas receptiveness to low-intensity, self-help approaches was mixed. Across the two sites, adolescents, parents, and teachers had no previous experience of self-help interventions, but they were broadly receptive to blended approaches that combined self-help materials with face-to-face relational and instructional support from a counsellor. Adolescents were amenable to digital self-help formats, notwithstanding limited availability of personal electronic devices (especially in Delhi). Parents and teachers were concerned about the misuse of digital technology and favored ‘off-line’ self-help formats. Further research is needed to examine how to reconcile competing stakeholder perspectives on digital mental health interventions, particularly with rapidly increasing Internet access among adolescents in India and other LMICs (International Telecommunication Union, 2017). Similarly, while group formats may provide an efficient way to increase the supply of evidence-based psychological interventions in low-resource contexts (Sharma *et al*. 2017), more research is needed to explore concerns regarding the acceptability of group-based delivery and scope for tailoring according to levels of need and other participant characteristics.

The scope for adolescents to participate autonomously and confidentially in SBMHSs also emerged as an area of disagreement between adolescent and adult respondents. Adolescents did not wish for parents or teachers to be directly engaged in any intervention, whereas these significant adults expected to be kept informed about adolescents' presenting difficulties and progress. Adolescents were wary about having their personal problems and feelings shared with other adults as a matter of course – and particularly in instances of sharing information about socially proscribed activities. Concerns about unwanted disclosure and resulting stigma were especially pronounced among adolescents in Goa, where SBMHSs had already been established in some of the collaborating schools. Previous reviews suggest that targeted sensitization activities, particularly focused on concerns regarding confidentiality and clarification of intended outcomes and methods, may be needed to increase uptake and sustained use of SBMHSs (Gulliver *et al*. 2010; Gronholm *et al*. 2018). More research is needed to formalize sensitization interventions that enable youth to feel safe and empowered when seeking psychological help. Parallel sensitization activities are needed to engage with adults who may play a crucial role in timely recognition of mental health problems and also positively influence help-seeking. Service programmers must also ensure that appropriate boundaries are established around information sharing; this may be particularly challenging in LMICs where statutory safeguarding frameworks and child protection responses are absent or poorly implemented (McTavish *et al*. 2019).

Notwithstanding divergent stakeholder views on specific formatting issues, there was broad consensus around the advantages of schools as a key setting for mental health services. SBMHSs have also been emphasized within India's current national mental health program and the RKSK and within the public health discourse more generally (Kumar *et al*. 2015; Sharma *et al*. 2017; Shinde *et al*. 2018; Roy *et al*. 2019). The current study therefore provides timely insights that are complementary to this evolving area of policy and service development, offering contextual evidence to shape more detailed specification of interventions and implementation plans.

Major strengths of the current study are the inclusion of a large sample, drawn from a wide range of stakeholders, and from two distinct sites in India. The study is limited by a relatively small number of participating parents and school principals in the total sample, and the use of FGDs for data collection with adolescents. Although the latter was an efficient method for obtaining qualitative data from a large adolescent sub-sample, it is possible that the group format limited the discussion of sensitive issues.

## Conclusions

Perspectives from multiple stakeholders have indicated that SBMHSs are an acceptable platform for delivering adolescent mental health care in India, with further consensus emerging around the critical importance of relational factors to engage adolescents in school counselling; the potential utility of problem solving as a core intervention element to address idiographic problems among help-seeking adolescents; and the need for targeted sensitization activities to support implementation efforts. Issues that touched on adolescent autonomy were more contentious, with adolescents preferring to participate in school counselling independently of other significant adults, but at the same time expressing doubts about the usefulness of ‘pure’ self-help without a guiding counsellor. Blended approaches to self-care (potentially involving a combination of digital and face-to-face formats) were seen as one way to reconcile preferences for developmentally and culturally appropriate therapeutic relationships, practical advice, instructional feedback, and personalized outcomes. Service developers and programmers should draw on these nuanced insights and other context-specific evidence along with the global evidence base in order to shape the future direction of school-based mental health services in India and other LMICs.

## Supplementary material

For supplementary material accompanying this paper visit http://dx.doi.org/10.1017/gmh.2019.16. click here to view supplementary material
