# Supplementary material for: Priorities and preferences for school-based mental health services in India: a multi-stakeholder study with adolescents, parents, school staff, and mental health providers
Source: Glob Ment Health (Camb). 2019 Aug 19;6:e18. doi: 10.1017/gmh.2019.16 (PMC6737585; doi:10.1017/gmh.2019.16)
Supplement: Supplementary file 1 [file gmhsup.zip › S2054425119000165sup002.docx]

**Additional File 2**

**A. Focus Group Discussion (FGD) Guide – School Going Adolescents**

Q) What are your thoughts about having a school based counselling program for adolescent mental health problems in your school?

*(Probe for benefits and concerns of such a program,*

*Probe if students would go to the counsellors themselves to seek help*

*Probe for problems that they would like to discuss with the counsellor*

*Probe for their thoughts about teachers referring the student to counsellor*

*Probe also for their thoughts about parents referring the student to counsellor)*

Q) How do you think counselling might help a student?

*(Probe for what would indicate a positive difference in their lives*

*Probe for outcomes related to mental health concerns – sadness, stress levels, fears and anxiety, anger and violent outbursts.*

*Probe also for outcomes related to attention and performance in class, studies and performance in exams, in extra-curricular activities, outcomes related to relationships with friends, parents, other students.*

*Probe for skills/ information they would like to get through the counselling program)*

Q) Counselling will be taking place at school. What are some potential concerns a student might have about that or about counselling in general?

*(Probe for feeling shy or embarrassed or concerned with other students and teachers knowing about them going to the counsellors.*

*Probe for concerns regarding sharing of information with parents.*

*Probe for missing a class or two for the same.)*

**Self-help, web based intervention:**

We would like to now know your thoughts on possibility of having a way to help young people learn skills on their own and to understand and solve their r problems. E.g., a workbook or an app can be provided to teach young people how to approach their problems systematically and a counsellor could guide them through it. It will include some exercises and homework related to solving problems.

Q) How do you think young people might respond to such a workbook?

*Probe for pros and cons of self-help; pros and cons of using a workbook; what would help in completing it?*

Q) What suggestions do you have to make such a workbook appealing to young people?

*(Probe about use of graphics, games, write-in activities, examples, vignettes, etc.)*

Q) What do you think might get in the way of using such a workbook?

*(Probe for forgetting workbook, not completing homework, feeling pressurised to complete the workbook as a part of curriculum, lack of tie at home to fill in the workbook. copying from friends’ workbook, etc.)*

Q) Do you have any thoughts on ways to make it easier for youth to use this workbook?

Q) How do you think the use of technology might enhance this workbook or improve the experience for young people?

*(Might have to explain that the tablet would be given through the school which will have to be returned in a good condition)*

Q) Do you or your friends use a smartphone/tablet? If yes, what do you use it for and what are your favourite apps or games on the phone or computer?

*(Probe for playing games, music, chatting, movies, watching videos etc.*

*Explore why they enjoy certain games, apps, websites better than others)*

Q) Do you know of any young people who have used technology to get help for stress or other concerns? If so, can you describe what they did?

*(Probe for google search, websites used, apps used)*

Q) How might parents, teachers, and/or counsellors support young people through the completion of this workbook? How might they be Unhelpful?

*(Probe for who would be preferred.*

*Probe for how and how much of the involvement by peers, parents and teachers would be desirable,*

*Probe specifically for what might not be preferable with regards to involvement of parents, teachers and peers for completion of workbook)*

**B. In-Depth Interviews and Focus Group Discussion guide for Mental Health Practitioners and School counsellors**

**Mental Health Problems in Young Persons**

Q) Please tell us from your experience, what are the most common mental health problems adolescents approach you with?

Q) What according to you are the major underlying factors that influence a young person’s mental health?

*(Probe for factors related to home, school, peers and individual - age, gender and SES based differences in the problems observed)*

Q) What strategies do you think the adolescents generally use for coping with these problems?

*(Probe for common coping strategies, the stage at which they approach mental health practitioners including school counsellors, openness to involve parents, teachers and peers in addressing these concerns, age, gender and socio-economic based differences observed)*

Q) How do the parents and teachers deal with such problems in young persons?

*(Probe for initiatives and actions taken by parents at home and teachers in schools including referral to the counsellor, professional)*

**School based services**

Q) Can you tell us how are the adolescents referred to you?

*(Probe for referral from self, parents and teachers – type of problems referred, types missed, conditions for referral to specialists, etc.)*

Q) What according to you are the outcomes of interest to students, teachers and parents from a school based service?

*(Probe for clinical outcomes, functional outcomes)*

Q) What are the specific strategies that you have found to be helpful to engage adolescents?

*(Probe for strategies for different types of problems, attracting and retaining attention, exercises, approach of the counsellor, activities conducted within schools to improve engagement of adolescents, involvement of peers, family, teachers in engagement, strategies for reducing in drop-out)*

Q) What are the common barriers that students face in accessing such a service in schools? What can be done to address them?

*(Probe for anticipated stigma, and actual discrimination faced by referred adolescents, issues from school pertaining to missing classes, or potential concerns from home)*

Q) What is the current role of parents, teachers and the school in the therapy process? What more would you have liked?

*(Probe for their involvement in counselling therapy, issues of confidentiality, etc.)*

Q) What do you think about providing a self-help treatment as an initial step to adolescents suffering from mental health issues as discussed above?

*(Probe for possible target problems, strategies, effectiveness in terms of outcomes for mental health and functionality, age of adolescents for whom it might be better, content, format – workbook/ app/ others, and delivery of the self-help, involvement of counsellor, parents and teachers)*

Q) Do you think designing an intervention using self-help would be acceptable and feasible in a school set-up?

*(Probe for acceptability to counsellors, teachers and adolescents)*

Q) How can the adolescent‘s engagement in self-help be enhanced?

*(Probe for specific engagement strategies relevant for self-help,*

*Probe for provisions for addressing emotional and behavioral problems, tailoring to the specific problems identified, age and gender)*

**C. In-depth interview guide for parents**

**Emotional and Behavioral Difficulties faced by Adolescents**

Q) What does the word ‘mental health of your child’ mean to you?

*(Probe – when would you say a child is mentally healthy*

*What would make you think that a child is having poor mental health)*

Q) What do you think are the most common mental health problems in adolescents?

(*Probe for problems like tension, stress, sadness, depression, anxiety or fearfulness and low confidence, concern over the way they look, exam stress, anger issues, destructive/disruptive behaviours, bullying, attention problems, learning problems, suicidal thoughts, substance abuse, drugs, etc.*

*Probe for causes like family problems, love problems, problems with peers, studies, etc.)*

Q) How do you think these problems impact the lives of the young persons?

*(Probe for - managing at school, at home and with friends?)*

Q) What are the factors that have an effect on the mental health of the young persons?

*(Probe for factors influencing mental health negatively*

*Probe for factors influencing positively*

*Probe also for the role of parents, home environment, school related-, individual related- and peer circle related- factors)*

Q) How do young persons of your child’s age generally deal with such problems?

*(Probe for coping mechanisms to address emotions, problems, and thoughts.*

*Probe for strategies to manage emotions and stress, use of alcohol, drugs, violent outbursts)*

Q) How do you think do the parents generally deal with such problems among their children? For example, what would a parent generally do if their child has started staying isolated and is not taking interest in activities that he/she would generally like, remains sad, dull or and has also started to show a decline in school performance be it marks, or participation in sports)

**School based services:**

Q) What are your thoughts on having a school based mental health program in this school?

*(Probe for anticipated benefits to children*

*Probe for anticipated challenges and concerns*

*Probe for strategies to address these challenges)*

Q) How do you think such a program would help the child?

*(Probe for outcomes related to mental health concerns – sadness, stress levels, fears and anxiety, anger and violent outbursts.*

*Probe also for outcomes related to scholastic performance, interpersonal relationships at home and with friends, general behaviour at home and at school, etc.)*

Q) What do you think would be the concerns of a parent about sending a child for counselling in school?

Q) Teachers might also be referring the students to counsellors and sometimes children themselves also see the counsellor for counselling. What would be a parent’s response regarding this?

*(Probe for acceptability and concerns regarding referral by teachers*

*Probe for requirement to inform the parent about the referral both for self and teacher referrals)*

Q) How can we involve parents in the program?

*(Probe for referral from parents and the problems they think should be referred to a counsellor)*

**Self help**

We would like to now know your thoughts on possibility of having a way to help young people learn skills on their own and to understand and solve their problems. A workbook will be provided to teach young people how to approach their problems systematically and a counsellor will guide them through it. It will include some exercises and homework related to solving problems.

Q) How do you think young people might respond to such a workbook?

*(Probe for pros and cons of self-help;*

*Pros and cons of using a workbook)*

Q) What suggestions do you have to make such a workbook appealing to young people?

(*Probe about use of graphics, colourful presentation, games, quizzes, write-in activities, examples, vignettes, etc.)*

Q) What do you think might be the problems associated with using the workbook?

*(Probe for forgetting workbook, not completing homework, copying or asking someone else to complete the workbook, reading and writing difficulties, time to complete the workbook at home, etc.)*

Q) Do you have any thoughts on ways to address these problems and make it easier for youth to use this workbook?

*(Probe for help from parents, teachers, peers to complete the workbook)*

Q) How do you think the use of technology might enhance this workbook or improve the experience for young people?

*(Might have to explain that the tablet would be given through the school which will have to be returned in a good condition)*

Q) Do you think children can use the smartphone or a tablet? What do the generally use it for?

*(Probe for playing games, music, chatting, movies, watching videos etc.*

*Explore why they enjoy certain games, apps, websites better than others)*

**D. Focus group discussion and In-depth interview guide for parents**

**Description of experience working with students with mental health problems**

Q) What does the term ‘mental health’ mean to you?

*(Probe for what are the first thing that comes to mind when you hear this term ‘mental health’? How would mental health of students be important to you as a teacher?)*

Q) What mental health problems seem to be the most common among adolescent students.

Q) What could be the underlying factors that influence a student’s mental health?

*(Probe for factors related to home, school, peers and person related)*

Q) In what ways do these problems affect the students?

*(Probe for impacts in school setting, at home and among peers)*

Q) How do teachers generally respond to a student with one of these problems?

*(Probe for existing school services for health and mental health.*

*Probe for teachers’ role in identification and referral,*

*Examples of actions taken for students identified with such problems in the past,*

*Probe also for difference in approach based on the type of problems (emotional, behavioural and learning), age and gender (boys and girls) of the adolescent*

*Probe for their observations on differences across the teachers’ responses)*

**Psychotherapy in schools**

Q) What are your thoughts on having a counselling program in school?

*(Probe for anticipated benefits and problems for school, teachers and students*

*(Probe for teachers’ role in identification and referral and contacting/informing parents)*

Q) How would students and parents respond to a referral by teachers to counsellors?

*(Probe for denial, feelings of shame, weakness, or punishment among students and parents*

*Probe for involvement of parents in the counselling and encouraging their involvement including referral)*

Q) For a student you referred, how do you think the counselling therapy would help?

*(Probe for specific outcomes related to emotional health, confidence,*

*Probe for functional outcomes like performance in studies, obedience, participation in sports, cultural activities, and attendance, attentiveness and behaviour in class, friends circle, etc.*

*Probe for specific information or skills the teachers thought students should receive through counselling)*

Q) To what extent are students likely to refer themselves for counselling?

*(Probe for problems most likely to be identified by students*

*Probe for parents’ response to their self-referral*

*Probe for how can parents be encouraged to refer students)*

Q) What do you think are the potential concerns and challenges about having counselling services in schools?

*(Probe for anticipated stigma, concerns regarding excusing students for counselling session, space constraint, challenges in teachers and parents involvement, others)*

Q) Can you suggest strategies to address these concerns.

*(Probe for additional strategies for ensuring that counselling services are used by the teachers and students)*

**Self-Help**

We would like to now know your thoughts regarding possibility of providing therapy that would help young people to understand their problems and develop skills to address these problems on their own. A workbook/app will be provided by the counsellor to students that explains the concepts of the therapy and provides homework and exercise for them to practice the skills to address their problems.

Q) How might the idea of self-help be perceived by your students?

*(Probe for pros and cons of self-help including acceptability by students, parents, compliance, anticipated effectiveness, etc)*

Q) How can the workbook be made more appealing to young people?

*(Probe for language, presentation using graphics, pictures, colours, stories, quizzes, etc)*

Q) What additional information should be given to the students that can help them in addressing their problems?

Q) What role, if any, do you see for teachers in supporting students to use the self-help materials?

*(Probe for parents’ role in supporting the use of self-help,*

*Probe for strategies to engage parents and increase their involvement in going through self-help materials)*

Q) What do you think of using tablets or smartphones to provide self-help materials instead of the workbook?

*Probe for what might be more preferred by students along with reasons*

*Probe for what might be more acceptable and feasible to the schools with reasons)*

Q) What might be the potential issues concerning use of workbooks and apps?

*(Probe for non-completion of workbooks, and difficulties in using the workbook or app Probe for barriers related to readability, familiarity with smartphones/apps, perceived stigma, potential interference with studies, concerns with parents’ acceptability, theft and misuse of tablets)*

Q) How do you think can these challenges be addressed?
