# Supplementary material for: Priorities and preferences for school-based mental health services in India: a multi-stakeholder study with adolescents, parents, school staff, and mental health providers
Source: Glob Ment Health (Camb). 2019 Aug 19;6:e18. doi: 10.1017/gmh.2019.16 (PMC6737585; doi:10.1017/gmh.2019.16)
Supplement: Supplementary file 1 [file gmhsup.zip › S2054425119000165sup001.docx]

Consolidated criteria for reporting qualitative studies (COREQ): 32-item checklist

| **No** | **Item** | **Description** | **Location in the manuscript (Section, pg no.)** |
| --- | --- | --- | --- |
| **Domain 1: Research team and reflexivity** |  |  |  |
| Personal Characteristics |  |  |  |
| 1. | Interviewer/facilitator | RP, MS, AS (Other interviewers involved are acknowledged in the manuscript.) | Methods, 9 |
| 2. | Credentials | RP: MPH  MS: MPH  AS: MPH  Other interviewers (acknowledged) held graduate, or Master’s degree or PhD in psychology or public health. | Title page, Methods, 9 |
| 3. | Occupation | RP, MS, AS were employed with the Public Health Foundation of India. RP was a PhD candidate at the time of conducting the study. | Title page |
| 4. | Gender | RP, MS, AS: Females; other interviewers who conducted FGDs included both males and females. | Methods, 7 |
| 5. | Experience and training | RP, MS and AS had acquired basic training on qualitative research in MPH program and have more than 3 years of research experience including qualitative research. RP also had 2 weeks training on qualitative research methods including analysis at Public Health Foundation of India before the study. | Title page, Methods, 9 |
| Relationship with participants |  |  |  |
| 6. | Relationship established | Yes | Methods, 7 |
| 7. | Participant knowledge of the interviewer | Participants were briefed about the purpose of the study and written information was also provided for both adolescents and parents. The necessary ethical approvals were received before commencement of study. | Methods, 7 |
| 8. | Interviewer characteristics | There are no potential sources of bias. | - |
| **Domain 2: study design** |  |  |  |
| Theoretical framework |  |  |  |
| 9. | Methodological orientation and Theory | Thematic content analysis | Methods, 9 |
| Participant selection |  |  |  |
| 10. | Sampling | Purposive | Methods, 7 |
| 11. | Method of approach | Researchers approached participants through announcements in school classrooms and trough visits to community based organisations working with the school going adolescents of the participating schools. | Methods, 7 |
| 12. | Sample size | 300 | Methods, 7, Table 1 |
| 13. | Non-participation | All potential participants identified themselves after learning about the research program agreed for participation. | Methods, 7 |
| Setting |  |  |  |
| 14. | Setting of data collection | 19 of 22 Focus Group Discussions (FGDs) with adolescents were conducted in schools, and the remainder 2 in community settings.  All teachers were interviewed in schools.  7 in-depth interviews (IDIs) with parents were conducted in school and 2 parents were interviewed in office of the Indian research organization.  All psychiatrists and psychologists were interviewed in their clinics.  4 School counsellors were interviewed in schools and 11 school counsellors were interviewed in their office. | Table 1 |
| 15. | Presence of non-participants | No. |  |
| 16. | Description of sample | A sample of 191 adolescents was drawn from 9 secondary schools in Delhi and 7 secondary schools in Goa, along with 75 teachers; 3 school principals, 9 parents, 5 psychiatrists, 2 clinical psychologists and 15 school counsellors were interviewed. | Methods, 7 and Table 1 |
| Data collection |  |  |  |
| 17. | Interview guide | Yes, the interview guide was prepared by the authors for the purpose of this study. | Methods, 9, Additional File 2 |
| 18. | Repeat interviews | No |  |
| 19. | Audio/visual recording | Yes, we audio-recorded all where permission was granted by participants, but 3 FGDs and 2 IDIs were not recorded | Methods, 9 |
| 20. | Field notes | Field notes were made during the FGDs and the IDIs | Methods, 9 |
| 21. | Duration | 45-60 minutes | Methods, 9 |
| 22. | Data saturation | Data saturation was discussed within the team by reviewing the field notes. | Methods, 9 |
| 23. | Transcripts returned | No | - |
| **Domain 3: analysis and findings** |  |  |  |
| Data analysis |  |  |  |
| 24. | Number of data coders | Four | Methods, 9 |
| 25. | Description of the coding tree | Deductive and inductive codes were developed. Related codes were ordered into meaningful categories to convey inter-related ideas. | Methods, 9 |
| 26. | Derivation of themes | Yes | Methods, 9 |
| 27. | Software | Nvivo, 11. | Methods, 9 |
| 28. | Participant checking | No. | - |
| Reporting |  |  |  |
| 29. | Quotations presented | Yes, quotations from participants were used to present the findings. The age and gender of participant and site are described for every quotation. | Results, 10-20 |
| 30. | Data and findings consistent | Yes | Results, 10-20 |
| 31. | Clarity of major themes | Yes | Results, 10-20 |
| 32. | Clarity of minor themes | Yes | Results, 10-20 |
